# Supplementary material for: Looking into Pandora's Box: The Content of Sci-Hub and its Usage
Source: F1000Res. 2017 Apr 21;6:541. [Version 1] doi: 10.12688/f1000research.11366.1 (PMC5428489; doi:10.12688/f1000research.11366.1)
Supplement: Supplementary file 1 [file f1000research-6-12270-s0000.tgz › 39baf3c8-ff51-4a82-b748-c74ef2257466.pdf]

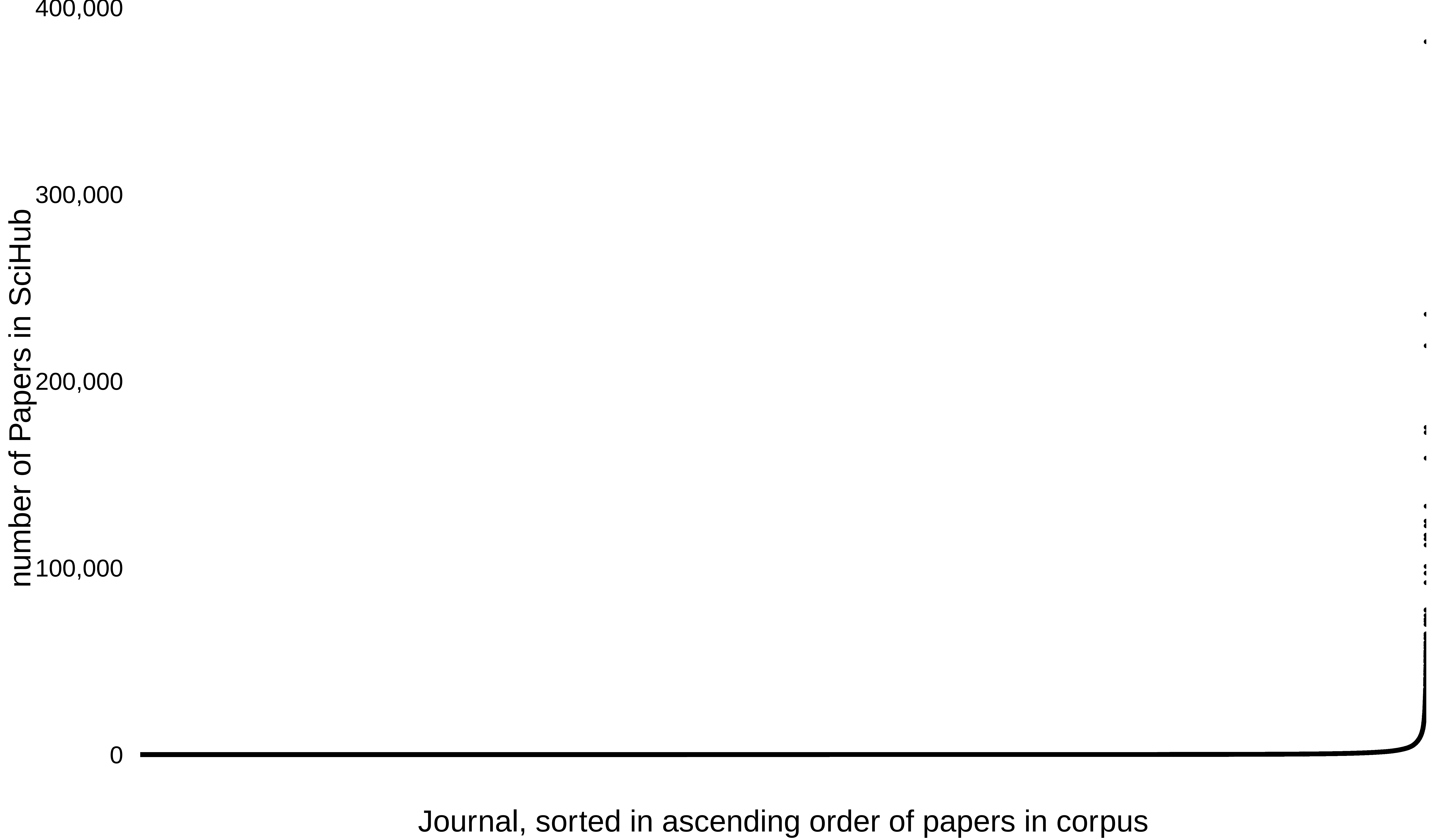

400,000
300,000
200,000
100,000
0
number of Papers in SciHub
Journal, sorted in ascending order of papers in corpus

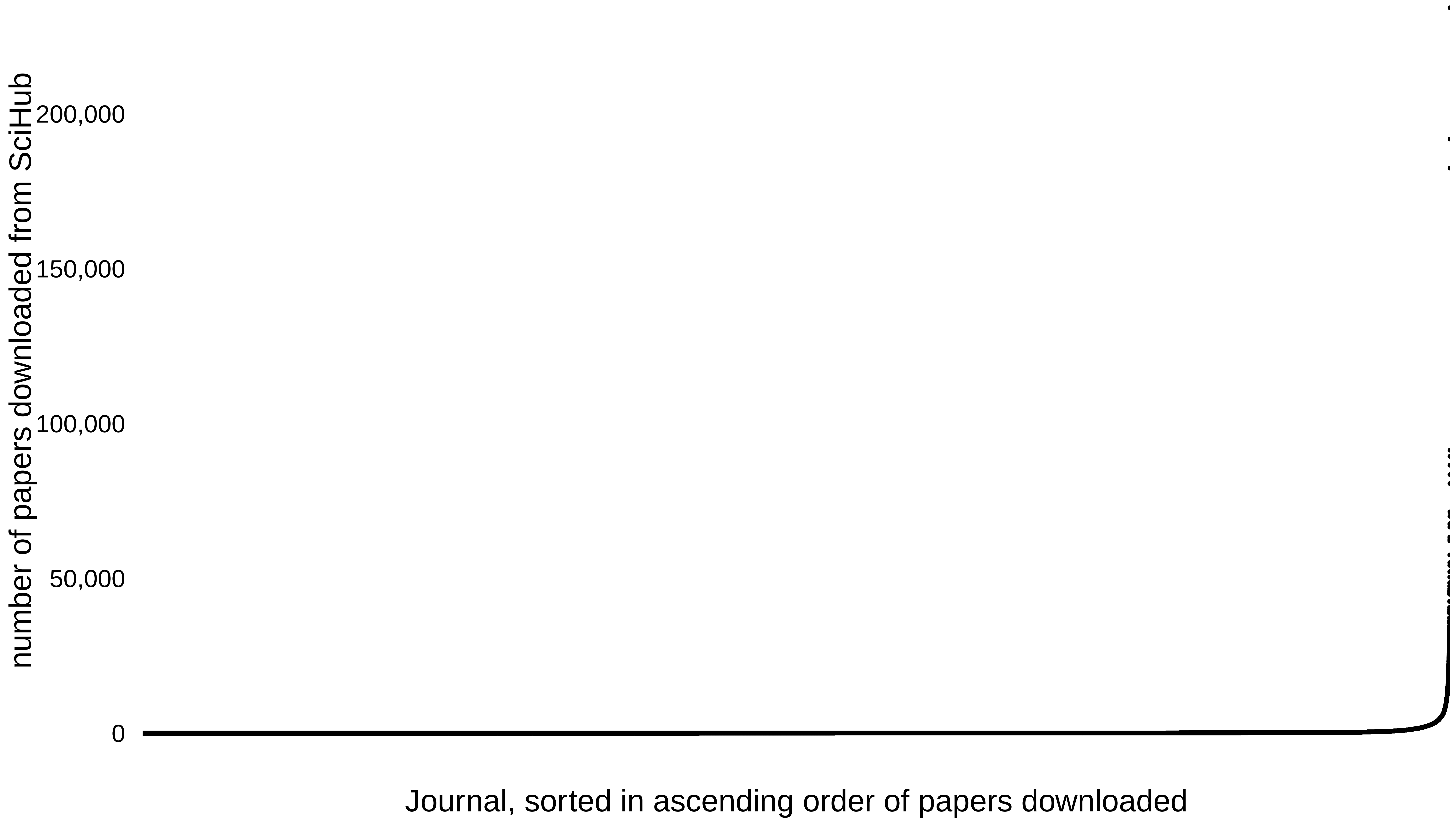
number of papers downloaded from SciHub
200,000
150,000
100,000
50,000
0
Journal, sorted in ascending order of papers downloaded
