# Supplementary figures and images for: Looking into Pandora's Box: The Content of Sci-Hub and its Usage
Source: F1000Res. 2017 Apr 21;6:541. [Version 1] doi: 10.12688/f1000research.11366.1 (PMC5428489; doi:10.12688/f1000research.11366.1)

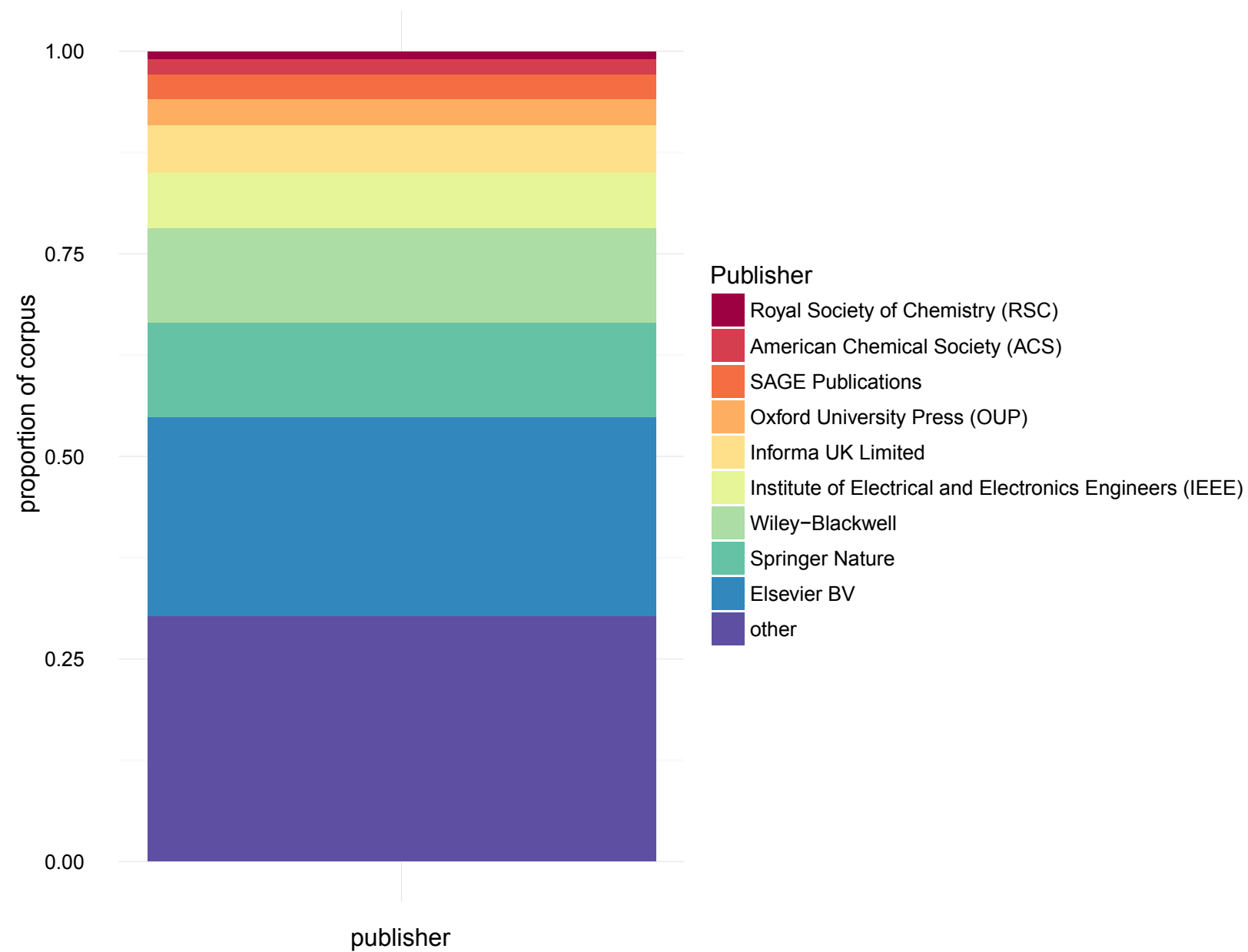

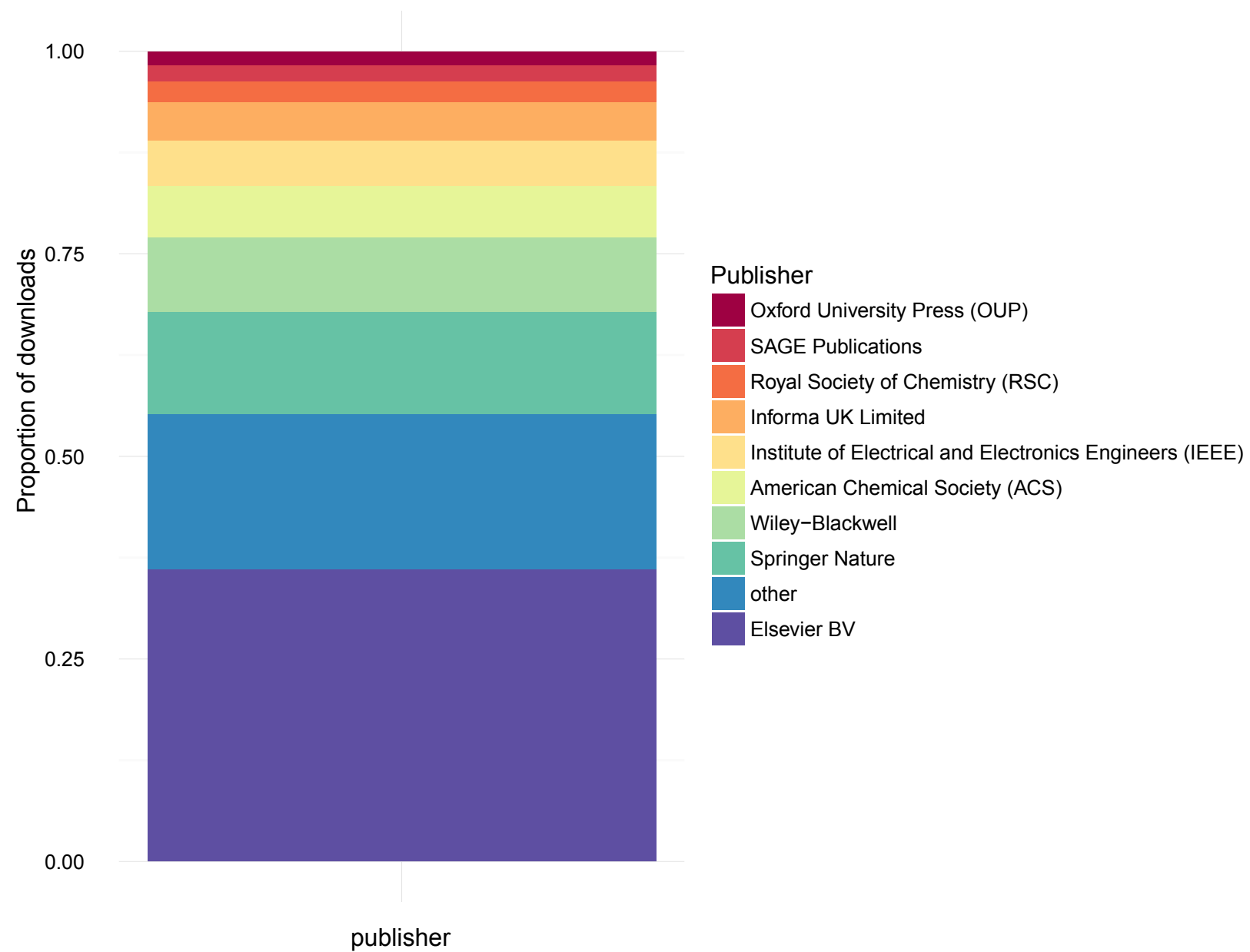

Supplement: Supplementary file 2 [file f1000research-6-12270-s0001.tgz › b4927bb9-3995-4a31-9ff1-5380fa9a01ae.pdf]
