# Supplementary material for: Looking into Pandora's Box: The Content of Sci-Hub and its Usage
Source: F1000Res. 2017 Apr 21;6:541. [Version 1] doi: 10.12688/f1000research.11366.1 (PMC5428489; doi:10.12688/f1000research.11366.1)
Supplement: Peer review history

# Peer review: Referee response for version 1

**DOI:** 10.5256/f1000research.12270.r22120

**Published:** 2017-05-12

**Authors:** Balázs Bodó

**Affiliations:** 1 Institute for Information Law, University of Amsterdam, Amsterdam, Netherlands

The analysis of shadow libraries usage data is not a trivial matter, and requires some caution, especially when someone tries to understand the processes that produce these usage numbers. The article is very modest in its aims, and hopes to present only a very basic analysis of the Sci-Hub usage, but I believe more could have been done in terms of the analysis, and more caution should have been used in when offering explanations.

 During the analysis, I think the logic of Sci-Hub allows us to distinguish between two processes: the one that *produces *the collection, and one that *consumes* the collection. Articles get into the Sci-Hub collection when someone bumps into a paywall, and turns to Sci-Hub to circumvent it. This means that the corpus of Sci-Hub is indicative of works that have limited accessibility. When analyzing the corpus, the distribution of publishers, and topics, one should look at it from this perspective, and check, for example the open access policies of the most highly represented publishers, or journals, and analyse the results not just within the sci-hub universe, but against the whole population of articles/journals/publishers/topics, including those with widespread open access policies.  

 The download numbers, on the other hand, represent the demand for an article. I would argue that articles with only 1 download only inform about the accessibility (someone met a paywall, and downloaded the article from sci-hub), while articles with more than 1 downloads actually suggest some things about the demand (how many individuals were interested in that article/discipline).

 On that note I missed the geographic analysis, especially as some data on the location of the download was also available in the original dataset.

 Regarding the interpretation of the data. I think the analysis in the Who’s reading? section is not substantiated by the data in any manner. On the contrary, while the data covers all downloads, across all the globe, the interpretation relies on a US census. I don't think that is appropriate. Local usage is structured and explained by local characteristics of higher education, research, and economy. One should not generalize a US explanation to the whole dataset.

 The analysis in the *Non solus *section is also misleading. It makes claims about the academic publishing space in general, while the sci-hub data is biased, as it only contains articles with accessibility problems. Articles, journals and publishers with no accessibility problems are probably missing from, or are heavily underrepresented in the dataset, thus one cannot come to any conclusion on the state of academic publishing. Take the case of PLOSone as an example, on why the current analysis is flawed.

 As a result, the validity of the overall conclusions is limited.

I have read this submission. I believe that I have an appropriate level of expertise to confirm that it is of an acceptable scientific standard, however I have significant reservations, as outlined above.

# Peer review: Referee response for version 1

**DOI:** 10.5256/f1000research.12270.r22123

**Published:** 2017-05-05

**Authors:** Jill Emery

**Affiliations:** 1 Portland State University, Portland, ON, USA

Bastian Greshake has done a good job in presenting his argument and providing supporting documentation. He may want to consider: Mark Ware's 2015 STM Report noted below ^1^ in regards to the research behaviour & motivation, as there may be information in this report that help further augment why SciHub is used & who is "reading". Greshake's graphs readily illustrate the points he is making regarding regarding the represented journals & publishers. His use of the publicly available data and noting both where the data is located and scripts used in order to perform his study lend to the transparency of his study. Lastly, these findings are of use and interest to librarians and information scientists as well as to product and resource developers looking to develop mechanisms to counter the "SciHub phenomena."

I have read this submission. I believe that I have an appropriate level of expertise to confirm that it is of an acceptable scientific standard.

## References

[1] WareM: The STM Report An overview of scientific and scholarly journal publishing. *STM*.2015; Reference source

# Peer review: Referee response for version 1

**DOI:** 10.5256/f1000research.12270.r22122

**Published:** 2017-05-02

**Authors:** Gabriel Gardner, Stephen McLaughlin

**Affiliations:** 1 California State University, Long Beach, CA, USA; 2 School of Information, University of Texas at Austin, Austin, TX, USA

In general, this is a clearly written and argued paper on a developing topic affecting the scholarly communications ecosystem. The author has engaged with much of the recent literature on the topic of which we are aware. The underlying data is freely available and thus possible to replicate. The quantitative analysis proceeds logically and is easy to understand. There are a few areas where we would like to see discussion expanded (noted below) though overall this paper is a very valuable contribution to the literature on this topic.

Specific Criticisms:

 The abstract brings up the question of who uses Sci-Hub and why. However, there is relatively little discussion of this in the paper. By our reading of the literature, the question has not been rigorously addressed to date. But some have taken steps toward an answer. Specifically Travis, (2016) is a data point worth discussing < http://www.sciencemag.org/news/2016/05/survey-most-give-thumbs-pirated-papers>. (The survey had a large response rate but should be viewed with the skepticism that would normally apply to any “open” internet survey.)

 The Supplementary Figures are worth incorporating into the text. S2, in particular, is an informative chart. It should be improved by matching the colors for each publisher in the legend. That is, “other” should appear as the darkest blue in both bars, rather than being assigned different shades of blue as it is presently. That will allow readers to observe the important differences easily.  

 Your methods section should include some additional discussion of what you mean by “expected number of downloads for each publisher.” You are using “expected” in a mathematical sense that diverges from the word’s everyday meaning, so you should spell this out for the reader.

 We find the use of the term “Black Open Access” in the discussion section puzzling. “Guerilla open access” is more widely used, as suggested by Google Trends < https://trends.google.com/trends/explore?q=%22black%20open%20access%22,%22guerilla%20open%20access%22>. Additionally, there are important issues of “respectability politics” to consider here; there are vocal OA advocates and practitioners who condemn Sci-Hub and do not want the OA movement to be associated with it or with copyright violation. Using the word “black” may be interpreted as implying that Sci-Hub is compatible with so-called green and gold OA publishing. Librarians in particular are loath to associate Sci-Hub with the OA movement, due to professional norms that often include upholding intellectual property restrictions on ethical grounds (e.g., < http://crln.acrl.org/content/78/2/86.full>,

 < https://thewinnower.com/papers/3489-signal-not-solution-notes-on-why-sci-hub-will-not-open-access>. On the other end of the spectrum, Sci-Hub’s supporters and sympathizers may object to negative connotations conjured by the term “black.” None of the above comments are meant to imply that your usage of “Black Open Access” is wrong. However, if you are going to use the less familiar term, you should explain why and note that this is a contested issue.

 In the Introduction section, your remarks on Sci-Hub’s legal status are well made, but another aspect of this is the fact that credential sharing is explicitly prohibited by many publishers (and some libraries) in their terms of use. This is worth mentioning. Elsevier’s and Wiley’s Terms are clear on this issue. < https://www.elsevier.com/legal/elsevier-website-terms-and-conditions> < http://onlinelibrary.wiley.com/termsAndConditions>

 Due to the ambiguous legality of copying factual and educational works under various copyright regimes, we prefer the terms “potentially illegal” or “likely illegal” when describing Sci-Hub’s activities. A recent ruling in India, for instance, suggests that Sci-Hub may not violate the law in that country.  

 < https://hughstephensblog.net/2016/09/27/the-indian-high-court-decision-on-delhi-universitys-copy-shop-a-pyrrhic-victory/>

 Also in the Introduction, the citation for the sentence discussing #icanhazpdf refers to Cabanac, 2015. However, #icanhazpdf is mentioned in that article only in passing. A more thorough analysis can be found in Gardner & Gardner, 2015. < http://eprints.rclis.org/24847/>

 Bodó deserves to be cited, but there are better sources on long-term changes in the academic publishing industry. Thompson (2005) is an especially good candidate. And Royster’s slides on the history of the OA movement [3] strikes us as insufficiently authoritative. Willinsky (2006) and/or Suber (2012) are potential alternatives.

 Under “Data Sources,” you should credit Elbakyan (not Hahnel) with releasing the list of DOIs in Sci-Hub. 

 < https://sci-hub.cc/downloads/doi.7z>

 < https://twitter.com/Sci_Hub/status/843546352219017218>

 ---

 Suber, Peter. 2012. Open Access. MIT Press Essential Knowledge Series. Cambridge, Mass: MIT Press.

 Thompson, John B. 2005. Books in the Digital Age: The Transformation of Academic and Higher Education Publishing in Britain and the United States. Cambridge, UK ; Malden, MA: Polity Press.

 Willinsky, John. 2006. The Access Principle: The Case for Open Access to Research and Scholarship. Cambridge, MA: MIT Press.

Minor corrections:

- Page 2, first sentence of 2nd paragraph: Change “was gotten from” to “was obtained from.”
- Page 2, last sentence of 2nd paragraph (and throughout): “peer-to-peer” is preferable to “peer2peer.”

- Page 2, first sentence of last paragraph: Change “publications is actually” to “publications are actually.”
- Page 2, last sentence of 3rd paragraph: Change “lead” to “led” (past tense).
- Page 2, first sentence of Data Sources (and throughout): Change “DOI” to “DOIs” for plural use.
- Page 2, second sentence of Data Sources: Change “downloads” to “download requests”
- Page 2, second sentence of Resolving DOIs: Change “meta data” to “metadata.”
- Page 2, second sentence of Results (and throughout): Insert comma after “i.e.”
- Page 3, first sentence of “Which Journals are Being Read?” and first sentence of “Are Publishers Created Equal?”: Change “at least a single paper downloaded” to clarify that you’re referring to the 6 months included in the log dataset.
- Page 3, first paragraph of the Discussion section: Change “large” to “largely.”
- Page 3, first paragraph of the Discussion section: “the whole corpus of Sci-Hub” implies you used the articles themselves. Change to “metadata for the whole corpus” or something similar.
- Page 3, second paragraph of the Discussion section: Change “more and more surpassed” to “more and more by.”
- Page 3, last paragraph: errant comma after ‘the long tail of’.
- Page 6, “Competing interests”: Change “SciHub” to “Sci-Hub.”
- Reference [1] should read “Balázs Bodó” instead of “Bodó Balázs.” “Bodó” is both his legal surname and his familiar name, so he occasionally flips the order.

We have read this submission. We believe that we have an appropriate level of expertise to confirm that it is of an acceptable scientific standard, however we have significant reservations, as outlined above.

# Peer review: Referee response for version 1

**DOI:** 10.5256/f1000research.12270.r22119

**Published:** 2017-04-25

**Authors:** April Hathcock

**Affiliations:** 1 Specialized Research Services, New York University , New York, NY, USA

This is a clear and well-researched paper on a very timely topic for science communication. I have just a few issues with some of the conclusions reached and with some of the literature represented in the review.

*So far it is not well studied how and why its users are accessing publications through Sci-Hub.*

 This isn’t necessarily true. The last year has seen a lot of articles pop up in the science communication and library literature about SciHub and the whys and hows of its use, including last year’s widely shared Science article by John Bohannon, which you briefly mention. This statement should be a bit tempered.

 Speaking of the whys of Sci-Hub, you discuss the founder’s description of how it is done but did not include any discussion from her about why she chose to develop the database. Her main occupation is as a scientist and she chose to develop SciHub because of being unable to access the literature in her field. I think that story is a compelling backdrop to your own research here.

 Again, Bohannon’s Science article from April 2016 “Who’s downloading pirated papers? EVERYONE,” gets very little mention in your paper. In any case, it certainly warrants a bit more discussion in your work. What did Bohannon do right in his analysis? Wrong? How does your work build on or diverge from his findings? In addition to Bohannon’s work, there have been a number of scholarly communication experts who have explored and written about they hows and whys of Sci-Hub usage, particularly in the library and information science field. I think a review of some of that literature would really help to ground your work.

*The analyses presented here suggest that Sci-Hub is used for a variety of reasons, by different populations.* You argue that your study shows that users use Sci-Hub for a “variety of reasons” but I don’t know that your research really supports that. Certainly you’ve shown what is being accessed and revealed interesting findings in terms of disciplinary, publisher, and publication date distribution, but your results can hardly be said to reveal the underlying motivations of users accessing materials from Sci-Hub. You posit some interesting theories that could explain the numbers you found (lack of access because of lack of well-funded institutional affiliation, etc.), but they are just that: theories. I’d be a bit more cautious in the conclusions you draw from your data, as interesting as they may be.

I have read this submission. I believe that I have an appropriate level of expertise to confirm that it is of an acceptable scientific standard.
